# His-bundle Pacing to Left Bundle Branch Pacing: Evolution of His-Purkinje Conduction System Pacing

**DOI:** 10.19102/icrm.2019.100504

**Published:** 2019-05-15

**Authors:** Pugazhendhi Vijayaraman

**Affiliations:** ^1^Geisinger Heart Institute, Geisinger Commonwealth School of Medicine, Wilkes Barre, PA, USA

**Keywords:** Biventricular pacing, cardiac resynchronization therapy, His-bundle pacing, left bundle branch area pacing, left bundle branch block

## Abstract

His-bundle pacing (HBP) constitutes an excellent alternative to right ventricular pacing. Recently, several studies have reported on the success and efficacy of HBP in patients with left bundle branch block requiring cardiac resynchronization therapy. Nonetheless, HBP may not always be feasible due to high capture thresholds or disease in the distal His bundle. The present report discusses the utility and feasibility of pacing in the left bundle branch area located distal to the site of conduction block.

## Introduction

His-bundle pacing (HBP) is an excellent physiologic alternative to right ventricular (RV) pacing in patients requiring ventricular pacing. HBP has been shown to be associated with a significant reduction in the combined endpoint of mortality, heart failure hospitalization, and upgrade to biventricular pacing in comparison with RV pacing in patients with bradycardia and indications for permanent pacemakers.^1,2^ Based on a systematic review of the available published literature on physiologic pacing, HBP was incorporated into the recently released 2018 American Heart Association/American College of Cardiology/Heart Rhythm Society guidelines on the evaluation and management of patients with bradycardia and cardiac conduction delay.^3^ HBP is recommended as a class IIa indication in patients requiring ventricular pacing who have a left ventricular ejection fraction of 36% to 50% and as a class IIb indication in patients with atrioventricular (AV) block at the level of the AV node. Recently, several studies have reported on the feasibility and efficacy of HBP in patients requiring cardiac resynchronization therapy (CRT) and HBP has emerged as a viable alternative to biventricular pacing (BVP).^4,5^ However, while HBP is feasible in the majority of patients requiring ventricular pacing, it may be more challenging to implement in some individuals due to the existence of high His-bundle capture thresholds or an inability to correct underlying His-Purkinje conduction disease. In the present report, the case of a patient in whom distal conduction system pacing was implemented to overcome the challenges associated with permanent HBP is discussed.

## Case presentation

**Figure 1:**
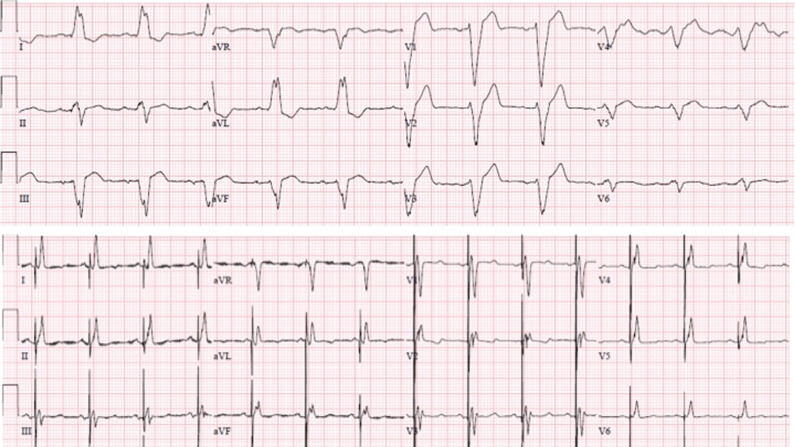
Twelve-lead electrocardiogram (ECG) at baseline and following BVP. The ECG demonstrates wide LBBB with a QRS duration of 190 ms. Following BVP, the QRS duration narrowed significantly to 128 ms.

A 71-year-old female with longstanding dilated cardiomyopathy, a left ventricular (LV) ejection fraction (LVEF) of less than 20%, New York Heart Association (NYHA) functional class III status, and left bundle branch (LBB) block (LBBB) with a QRS duration of 190 ms had undergone CRT utilizing BVP about 10 years prior to presenting for the current case **(Figure 1)**. Before her CRT operation, the patient had been on stable medical therapy, taking metoprolol 50 mg twice daily, lisinopril 20 mg daily, and furosemide 40 mg twice daily. Over the next two years, her LVEF had improved to 55% and remained stable, with an improvement in her NYHA functional status to class I. Her furosemide was subsequently discontinued. During the initial implantation, the LV lead (Acuity^®^ Spiral unipolar lead, model no. 4591; Boston Scientific, Natick, MA, USA) was implanted in a small- to medium-size lateral venous branch (excellent QLV), but with a capture threshold of 2 V at 0.5 ms. During follow-up, her LV capture threshold had steadily increased, likely as a result of proximal migration of the spiral-shaped lead. She underwent a generator change in 2012 due to battery depletion, at which time her LV capture threshold was 3.5 V at 1 ms. In 2018, she developed symptoms of exertional dyspnea, intermittent loss of LV capture at 6 V at 1.5 ms, and battery depletion and was referred for possible HBP.

**Figure 2:**
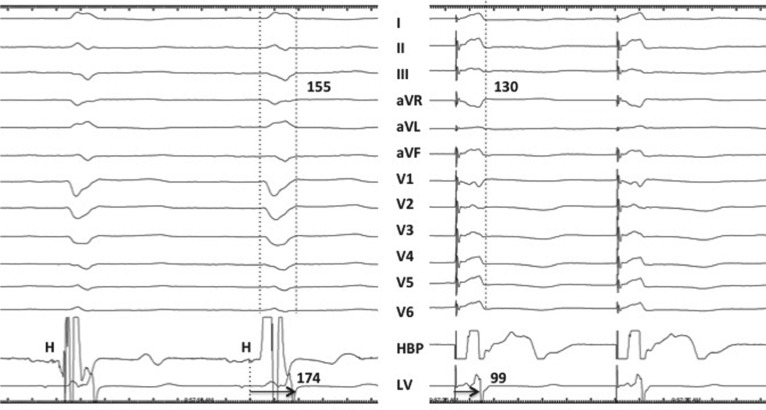
Twelve-lead ECG and intracardiac electrograms from the HBP lead and the LV lead in the coronary vein branch are shown at a sweep speed of 100 mm/sec. Following 10 years of BVP, the QRS duration in LBBB had decreased to 155 ms, suggesting LV remodeling. During nonselective HBP, the QRS narrowed to 130 ms. The His (H)-to-LV interval decreased significantly from 174 ms to 99 ms, with HBP suggesting LV resynchronization.

**Figure 3:**
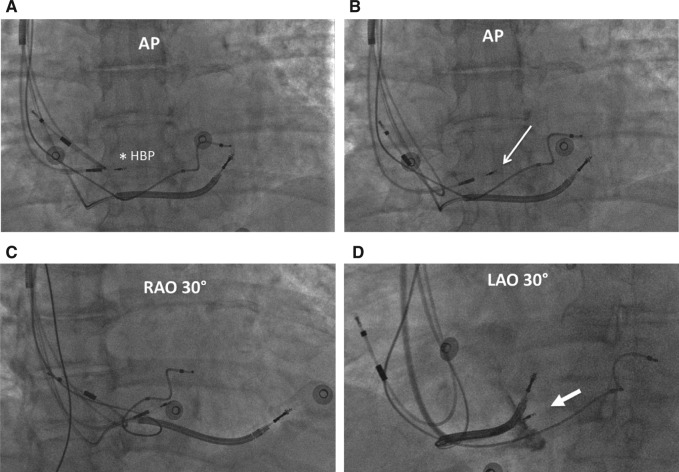
Fluoroscopic views of the LBBAP lead. **A:** Anteroposterior (AP) view of the lead at the His-bundle location (asterisk). **B:** The fluoroscopic location of the LBBAP lead at the initial site of fixation is located about 1 cm distally and inferiorly (arrow). **C:** The LBBAP lead location in a right anterior oblique 30° projection is shown. **D:** In a left anterior oblique 30° projection, contrast is injected to demonstrate the penetration of the lead body into the ventricular septum (block arrow). AP: anteroposterior; HBP: His-bundle pacing; LAO: left anterior oblique; RAO: right anterior oblique.

**Figure 4:**
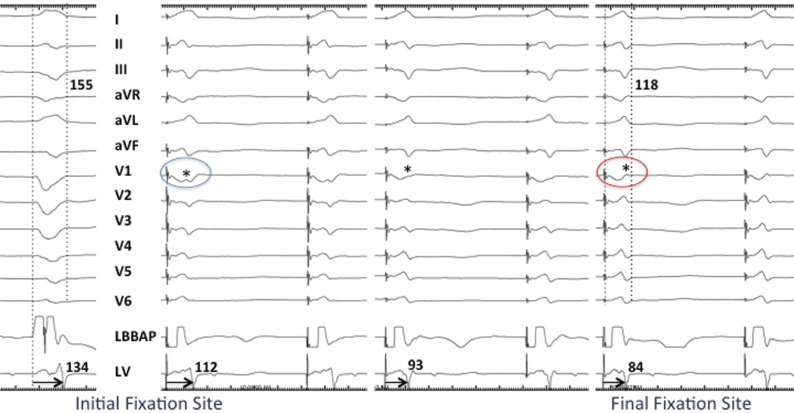
Electrical resynchronization during LBBAP. Twelve-lead ECG and intracardiac electrograms from the LBBAP lead and LV lead in the coronary vein branch are shown at a sweep speed of 100 mm/sec. Pacing from the RV septum before fixation demonstrates a “W” pattern with a notch (*) in the nadir of the QS complex in lead V1 (blue circle). As the lead was advanced progressively deep into the ventricular septum, the notch in V1 moved upwards toward the end of the QRS, resulting in a RBBB pattern. Simultaneously, the QLV was shortened significantly from 134 ms at baseline to a stimulus-to-LV interval of 84 ms at the final fixation site, along with the occurrence of narrowing of the QRS from 155 ms to 118 ms, suggesting excellent electrical resynchronization.

**Figure 5:**
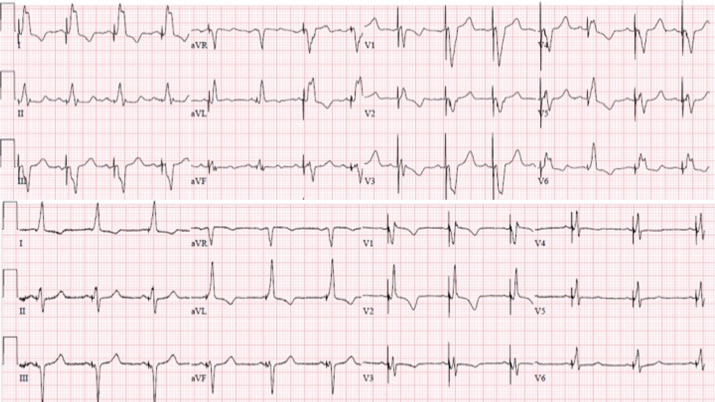
Twelve-lead ECG pre- and post-LBBAP. The top panel shows BVP with intermittent loss of LV capture. The bottom panel shows the QRS morphology during LBBAP.

Following explantation of the defibrillator, left axillary venous access was obtained and a 7-French sheath was placed. A C315 His sheath (Medtronic, Minneapolis, MN, USA) was advanced over a guidewire and placed in the tricuspid annular region. Using the SelectSecure 3830 pacing lead (Medtronic, Minneapolis, MN, USA) and unipolar mapping, the distal His-bundle location was identified. The His–ventricular (HV) interval was 40 ms at this location. During pacing at an output of 5 V at 1 ms, there was significant narrowing of the QRS duration to 130 ms. Of note, there was also a significant decrease in the His to LV timing as measured in the coronary sinus pacing lead of from 174 ms to 99 ms, corresponding to a change in QLV (the interval from the onset of the QRS complex to the negative deflection in the LV electrogram) **(Figure 2)**. However, following lead fixation, the pacing threshold to correct the LBBB was noted to be high at 2.5 V at 1 ms. Using the fluoroscopic HBP location as a reference, the sheath and lead were moved about 1 cm toward the RV apex, slightly posteriorly (inferiorly) **(Figure 3)**. Unipolar pacing at this location resulted in the QRS morphology of a “W” pattern in lead V1, where a notch was noted at the nadir of the QS complex **(Figure 4)**. The lead was rotated passively, while monitoring the unipolar pacing impedance and QRS morphology in leads V1 and V2. The pacing impedance progressively increased from 450 Ω to 780 Ω and the notch in the nadir of the QS complex in lead V1 moved up and toward the end of the QRS, resulting in a right bundle branch block (RBBB) morphology **(Figure 4)**. Contrast injection through the sheath in a left anterior oblique 30° projection revealed the depth of the lead to be about 5 mm in the septum **(Figure 3)**. The lead was advanced a few millimeters deeper by two additional rotations. At the final site, the paced QRS duration decreased from 155 ms at baseline to 118 ms **(Figure 5)**. Pacing threshold at this site was 0.5 V at 0.5 ms, R-wave amplitude was 14 mV, and pacing impedance was 750 Ω. This site was accepted, the sheath was removed, and the lead was anchored to the pectoral fascia. The previously implanted coronary sinus lead was capped and the deep septal LBB area pacing (LBBAP) lead was connected to the LV port of a new biventricular implantable cardioverter-defibrillator.

During follow-up, the patient felt significantly improved and her functional status returned to NYHA class I. A repeat echocardiogram performed at three months later revealed normal LV size and systolic function and an LVEF of 60% with no ventricular dyssynchrony. Her LBBAP lead threshold remained stable at 0.4 V at 0.5 ms.

## Discussion

CRT utilizing BVP has become a well-established option for the management of patients with cardiomyopathy, reduced LVEF, and LBBB. Super-response to CRT can occur in up to 30% of patients, especially in women with nonischemic cardiomyopathy.^6^ The patient described in this report was a classic super-responder to BVP with normalization of LV function along with significant structural (ie, decrease in LV end-diastolic diameter from 62 mm at baseline to 42 mm following BVP) and electrical remodeling. Prior to BVP, her QRS duration in LBBB was 190 ms, which had decreased to 155 ms at the time of lead revision 10 years later. Review of her initial procedural report described a posterior venous branch with high capture thresholds and phrenic nerve stimulation, limiting her options for coronary sinus lead placement. Traditional alternative options for BVP include epicardial LV lead placement via thoracotomy or a video-assisted thoracoscopic procedure. Other approaches include placing the lead across the interatrial or interventricular septum into the LV endocardium or using the novel WiSE-CRT system (EBR Systems, Sunnyvale, CA, USA) incorporating an LV endocardial microelectrode powered by ultrasound energy.^7,8^ Of course, these options are associated with technical challenges and additional procedural risks as well as a potential need for long-term antithrombotic therapy.

**Figure 6:**
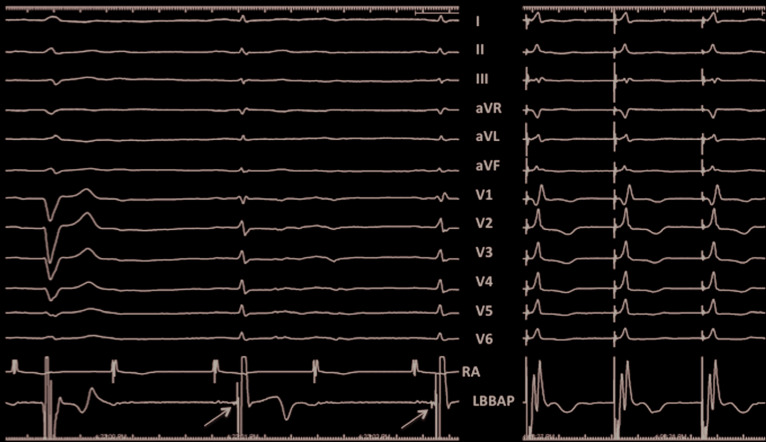
Recording of LBB potential. Twelve-lead ECG and intracardiac electrograms from the right atrial and LBBAP leads in a different patient with LBBB and intermittent 2:1 AV block with narrow complex at a sweep speed of 50 mm/sec. While no potentials are seen in the LBBAP lead during LBBB, a sharp potential 20 ms pre-QRS is seen (arrow) when conduction occurs via the left bundle, resulting in a narrow complex. RBBB morphology during unipolar pacing from the lead tip is shown on the right panel. Used with permission from: Vijayaraman P, Huang W. Atrioventricular block at the distal His bundle: electrophysiological insights from left bundle branch pacing. *HeartRhythm Case Rep.* 2019 Jan 23. In press.

Recent reports have demonstrated that the application of HBP in patients with typical LBBB and nonischemic cardiomyopathy can result in normalization of the LVEF in more than 80% of patients.^9^ However, in about 25% of patients with LBBB, HBP may not be able to correct the conduction abnormalities with an acceptable capture threshold. In the current patient, HBP resulted in the correction of LBBB, albeit at high capture thresholds, so it was not accepted as a feasible treatment option. It has recently been demonstrated that pacing the LBB distal to the site of conduction block in the His bundle is feasible and associated with excellent pacing thresholds.^10^ This is not technically challenging or associated with high morbidity as compared with the alternative approaches discussed above. In patients with complete LBBB, Purkinje potentials may not be recorded despite the lead being placed at the LBB area. However, if HBP results in the correction of LBBB, then potentials may be recorded, confirming the lead to be in the LBB area. Vijayaraman et al. recently demonstrated that it is possible to record the LBB potentials in patients with LBBB when conduction occurs through the LBB **(Figure 6)** or if an escape beat originates from the LBB.^11^

LBBAP was easily achieved in the present patient using the same delivery sheath and lead employed for HBP. While an LBB potential was not recorded in this case, pacing from this site resulted in a significant narrowing of QRS duration from 155 ms to 118 ms, suggesting engagement of the conduction system. More importantly, the local activation in the previously implanted LV lead as measured by the QLV interval served as an excellent surrogate for LV activation. During LBBAP, the stimulus-to-LV interval decreased significantly to 84 ms versus the baseline QLV of 134 ms, confirming the existence of electrical resynchronization by rapid activation through the Purkinje network **(Figure 4)**. Early experiences with LBBAP in patients with dilated cardiomyopathy and LBBB suggest remarkable electrical resynchronization associated with excellent clinical and echocardiographic response.^12^ LBBAP is correlated with excellent capture thresholds as compared with HBP or LV pacing via coronary sinus branches. In addition to being able to achieve a low and stable capture threshold, LBBAP is also an attractive option for pacing in patients with infranodal conduction disease while still maintaining LV synchrony.^11^ The long-term implications of LBBAP need to be carefully studied for a better understanding of both clinical outcomes and safety concerns. Nonetheless, LBBAP represents a natural evolution of His-Purkinje conduction system pacing to overcome the challenges posed by the current limitations of HBP.
